# Mapping the global literature output on nocturia: A bibliometric and visualized analysis

**DOI:** 10.1097/MD.0000000000029455

**Published:** 2022-08-05

**Authors:** Tauseef Ahmad, Muhammad Khan, Basem Al-Omari

**Affiliations:** a Department of Epidemiology and Health Statistics, School of Public Health, Southeast University, Nanjing 210096, China; b Vanke School of Public Health, Tsinghua University, Beijing 100084, China; c Department of Biotechnology and Genetic Engineering, Hazara University, Mansehra, 21120, Khyber Pakhtunkhwa, Islamic Republic of Pakistan; d Department of Epidemiology and Population Health, College of Medicine and Health Sciences, Khalifa University, Abu Dhabi, United Arab Emirates; e KU Research and Data Intelligence Support Center (RDISC) AW 8474000331, Khalifa University of Science and Technology, Abu Dhabi, United Arab Emirates.

**Keywords:** bibliometric analysis, HistCite™, nocturia, nocturnal polyuria, VOSviewer, Web of Science Core Collection

## Abstract

**Purpose:**

This study aims to facilitate researchers’ and clinicians’ understanding of research frontiers and trends in nocturia. It explores the scientific research outcomes and key bibliometric indices and plots global research on nocturia.

**Methods:**

A bibliometric retrospective study was designed, and an online search was conducted in the Web of Science Core Collection database using the potential search keywords related to nocturia in the title field with some specific filtration. HistCite™ and VOSviewer software for windows were used to analyze the data obtained for authors, journals, countries, institutions, keywords, and visualization mapping.

**Results:**

The initial search retrieved 1479 hits. A total of 1445 publications were included in the final analysis. Of these, 43.53% were published as articles. The most studied area in nocturia is urology nephrology. The most productive year was 2019 (n = 121, citations = 335), and the most prolific author, both in terms of publications (n = 97) and citations (1658) was Weiss JP. The most cited journal in nocturia research was the Journal of Urology (n = 293, citations = 3050). The most widely used keyword in nocturia publications was nocturia (n = 1249). Visualization mapping shows that the USA was the most influential and highly cited country in nocturia research.

**Conclusion:**

This study showed that there has been an increasing research trend in nocturia over the past few years. The current findings provide important empirical evidence for researchers, clinicians, and physicians to understand research frontiers and trends, achievements, collaborative networks, and hotspot research topics in the research field of nocturia.

## 1. Introduction

Over the last decade, nocturia (nocturnal polyuria) has become one of the most popular topics in the field of urology.^[1]^ Nocturia is one of the most common and troublesome urine-related symptoms.^[2]^ It can be caused by various medical complications and conditions, including renal tubular dysfunction, obstructive sleep apnea, cardiovascular disease, behavioral patterns, and diuretic medication.^[3]^ In such conditions, the patients wake up at night from sleep two times or more to urinate.^[4]^ The etiological factors of nocturia may vary by age, and the prevalence increases with age. By the age of 70 years, 62% of women and 59% of men wake up at least twice per night to urinate.^[5,6]^ In addition, behavioral and environmental factors, cardiovascular disease, diabetes mellitus, obesity, psychiatric problems, and sleep disorders have been suggested to be associated with nocturia.^[7,8]^ In the United States of America (USA), it affects an estimated 50 million people. The number of people diagnosed with nocturia is 10 million, of which only 1.5 million people receive specific therapy for nocturia.^[2]^

The impact of nocturia is associated with sleep fragment and sleep status rather than natural voiding.^[9]^ Sleep fragmentation coupled with nocturia can lead to reduced quality of life (QoL), reduce productivity at work, mood disturbance, and overall health problems with falls and fractures.^[10]^ Due to these severe consequences, the complaint of notaria should be a critical part of the clinical evaluation of patients’ urinary lower tract symptoms.

Nocturia is highly prevalent, occurs in both genders,^[11]^ and presents to general physicians and specialists. Although patients may present with lower urinary tract symptoms to their urologists, they may also present to the gynecologist, geriatrician, neurologist, sleep expert, endocrinologist, and/or general practitioner.^[12]^ For this reason, practitioners across all disciplines should follow a clinical algorithm, as described in the literature,^[13,14]^ to aid in making an accurate diagnosis and initiate an appropriate course of action. Recognizing the burden of the problem has been fundamental in this shift in understanding nocturia and the development of the multifactorial theory of causation.^[15]^

Therefore, it is important that clinicians, physicians, and health care professionals understand the etiology, associated factors, effective diagnostic methods, current achievements, and research trends in nocturia.^[16]^ Most importantly, understanding scientific research outcomes, recent trends, interventions, and control and preventive strategies for diseases and health complications can improve public health. In recent years, bibliometric studies have gained great attention in various disciplines, including medicine. Bibliometric analysis is a quantitative approach to scrutinizing the impact of scientific literature.^[17]^ Such studies are of great importance, as they not only provide comprehensive bibliometric indices but also determine hot spot research and future trends. This study aims to explore the scientific research outcomes, key bibliometric indices, and plot the global research on nocturia to assist healthcare professionals and researchers in understanding the latest research achievements and global trends.

## 2. Methods

### 2.1. Study design

A bibliometric retrospective study was designed.

### 2.2. Searching database and strategy

**Figure 1.**
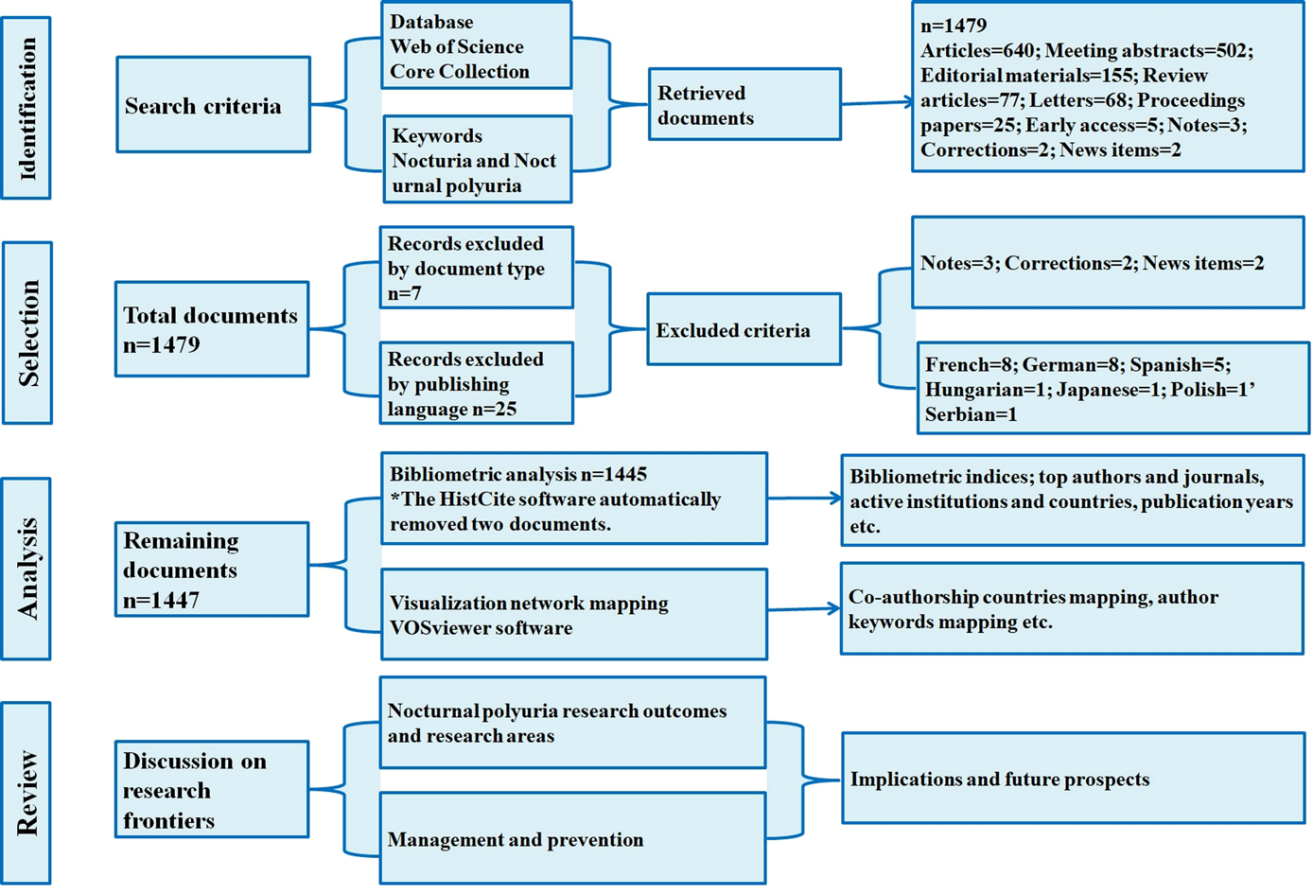
Publication selection and flow chart of the research framework.

An online search was conducted in the Web of Science Core Collection (WoSCC) database hosted by Clarivate Analytics.^[18]^ The Web of Science database is a widely used database in bibliometric analyses.^[19,20]^ The selected search database was accessed through the online library portal of Southeast University, China (time frame of the published documents on nocturia from 1920 to 2021). The search was conducted in August 2021 using the following search terms: “nocturia” OR “nocturnal polyuria” in the title field. The documents were refined by document types and publication language, as shown in Figure 1. The search was performed using the Boolean search strategy.^[21]^

### 2.3. Data extraction

All the data used in this study were downloaded independently by the first author from the WoSCC database. To facilitate data extraction and analysis, the data were downloaded in two formats: comma-separated values (CSV) and plain text. Self-designed data sheet was used for data extraction. Quarries related to document inclusion and data extraction were discussed and resolved with the help of other co-authors. The downloaded data were evaluated for several attributes, such as title, name of authors, journals name, year of publication, document types, keywords, institutions, countries, and top-cited studies.

### 2.4. Data analysis and visualization mapping

The data were exported into Microsoft Excel 2016. The values were presented as frequencies and percentages. The required graphs were generated using the OriginPro 2021. HistCite™ software was used to analyze key bibliometric indices.^[22]^ Visualization mapping was performed using VOSviewer software version 1.6.17.^[23]^ The dataset in plaintext format was exported into VOSviewer to plot the data for co-authorship countries’ network visualization mapping. The country with zero total link strength was excluded, and the cluster size was fixed at 5. After plotting the data, a total of six clusters were formed, and each color represented a different cluster. The maximum cluster size was 10, whereas the minimum cluster size consisted of six items. The stronger the collaboration between the two countries, the thicker the line. The higher the weight, the larger the label or node. The exported data were then processed for co-occurrence author-keyword network visualization mapping. The minimum number of occurrences of a keyword was selected as five. Eight clusters were formed.

## 3. Results

**Figure 2.**
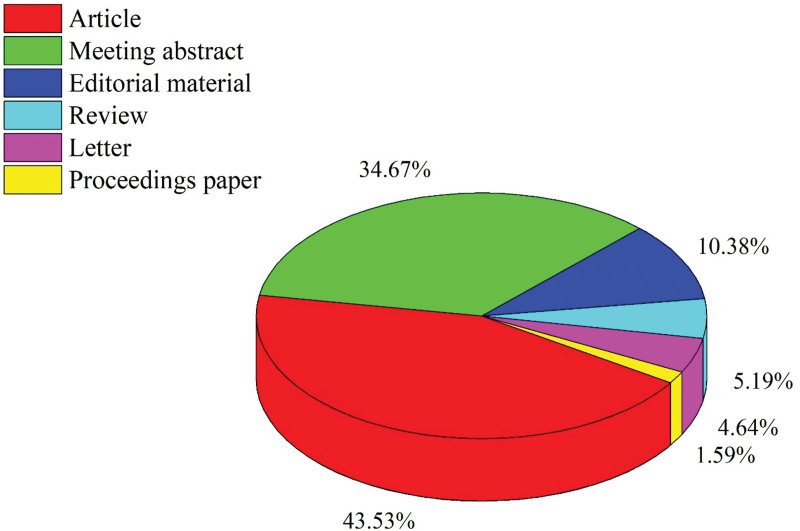
Document types of publications in nocturia.

**Figure 3.**
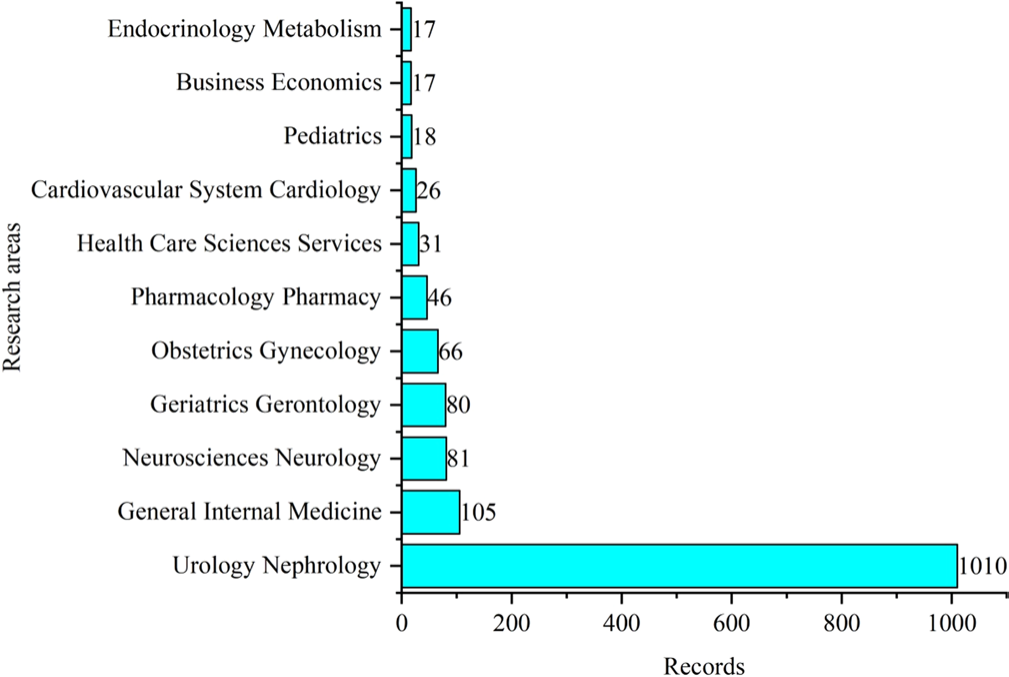
Top 10 most studied research areas in nocturia.

**Figure 4.**
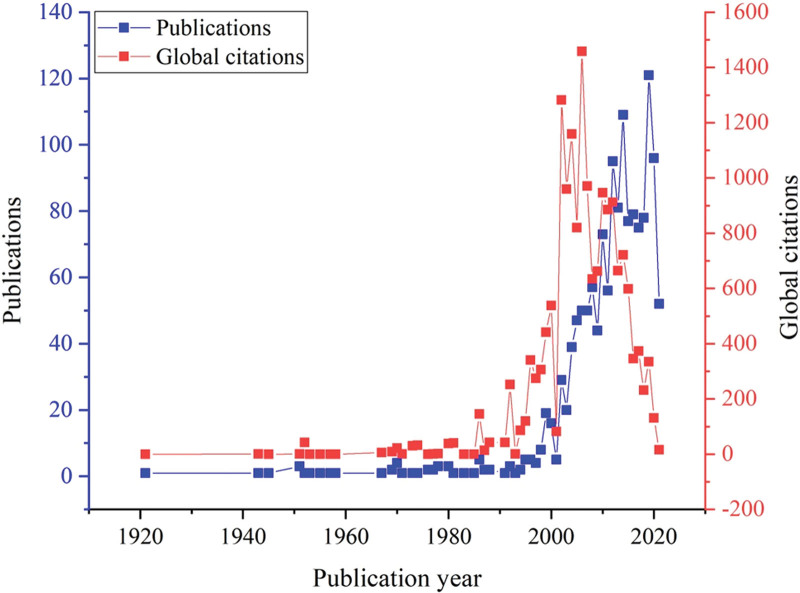
Year of publications and global citations of published documents on nocturia.

**Figure 5.**
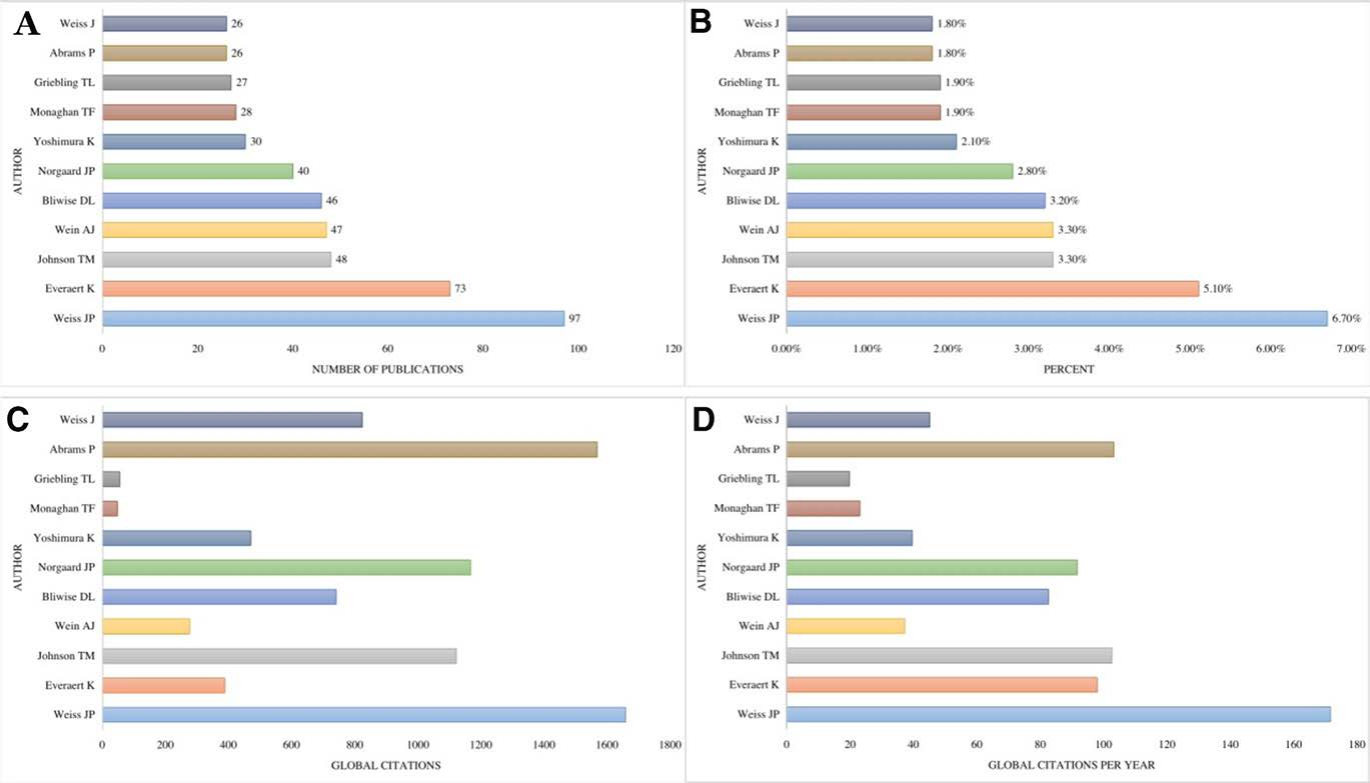
Top ten most prolific authors in nocturia research based on publications; (A) number of publications (B) percent (C) global citations (D) global citations per year.

**Figure 6.**
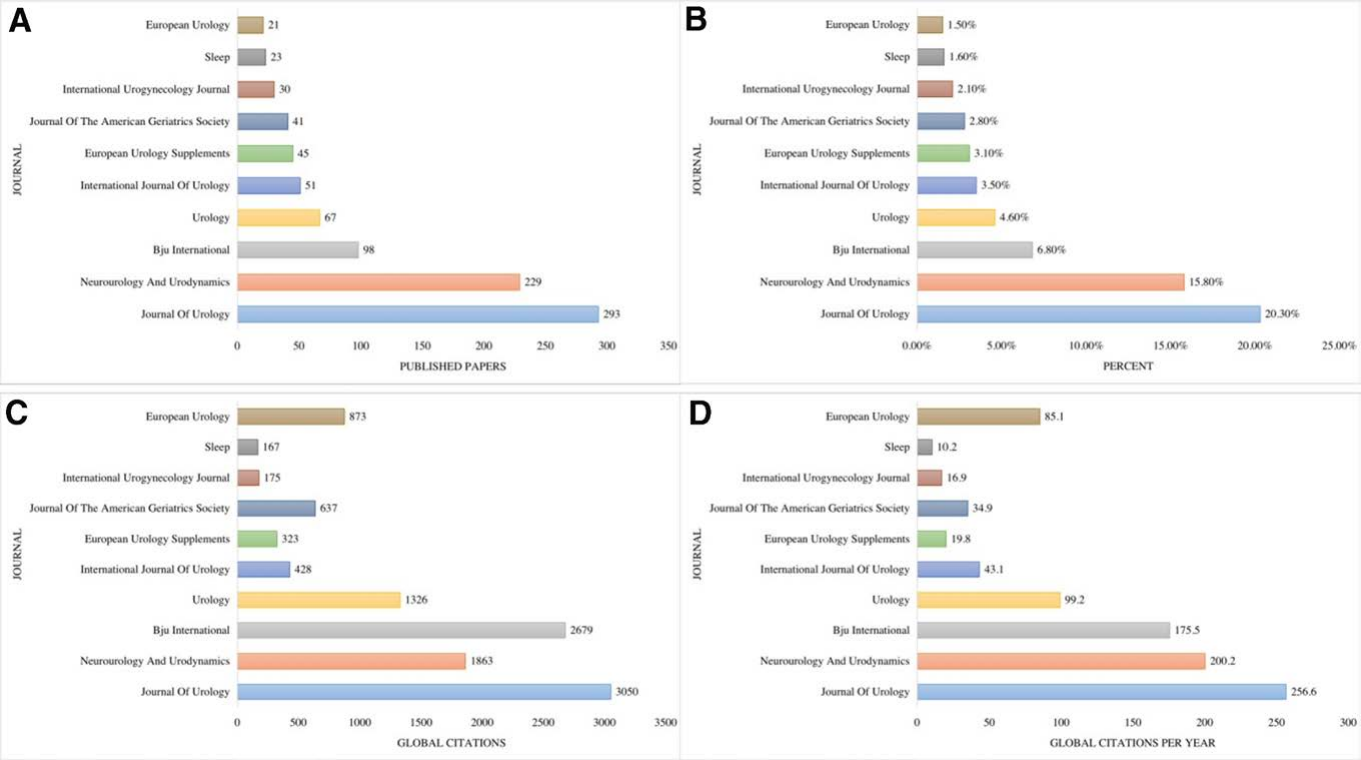
Top 10 journals in nocturia research; (A) total number of published papers (B) percent (C) global citations (D) global citations per year.

**Figure 7.**
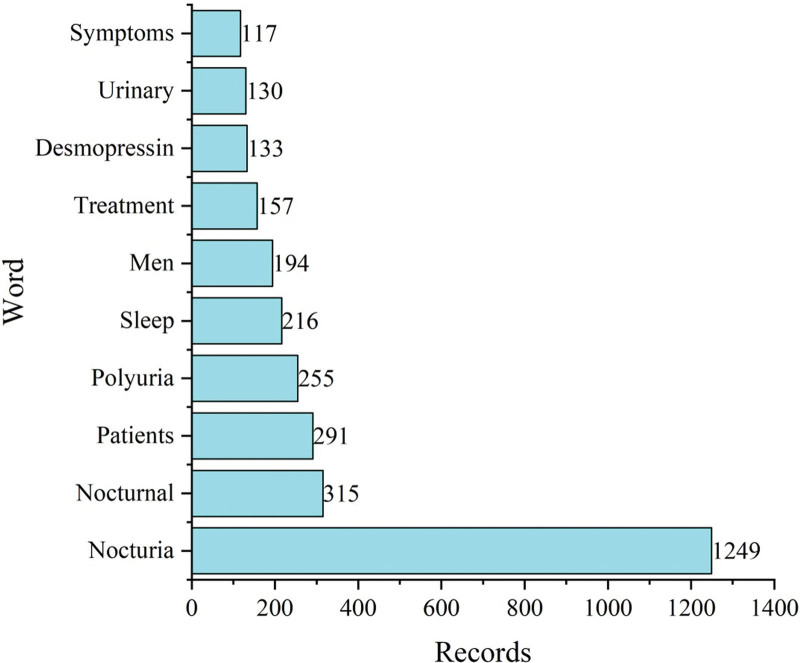
Top 10 most frequently used keywords in nocturia research.

**Figure 8.**
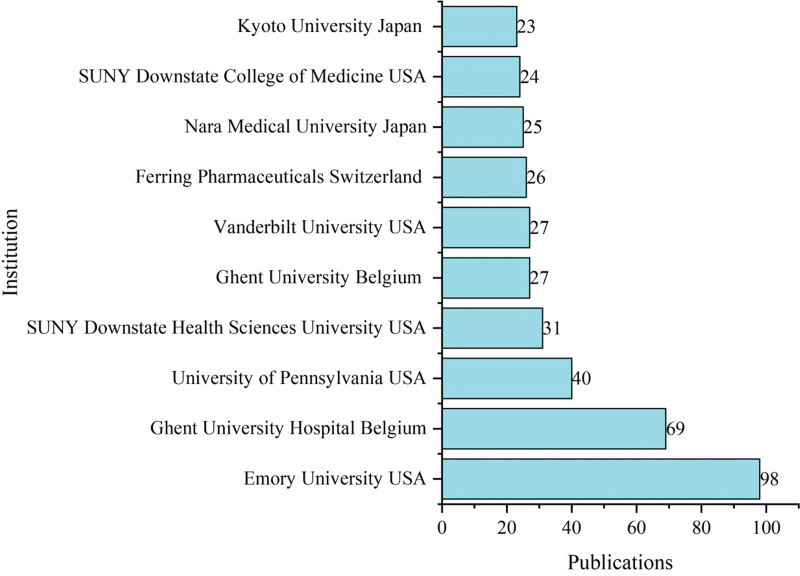
Top 10 most active institutions in nocturia research.

**Figure 9.**
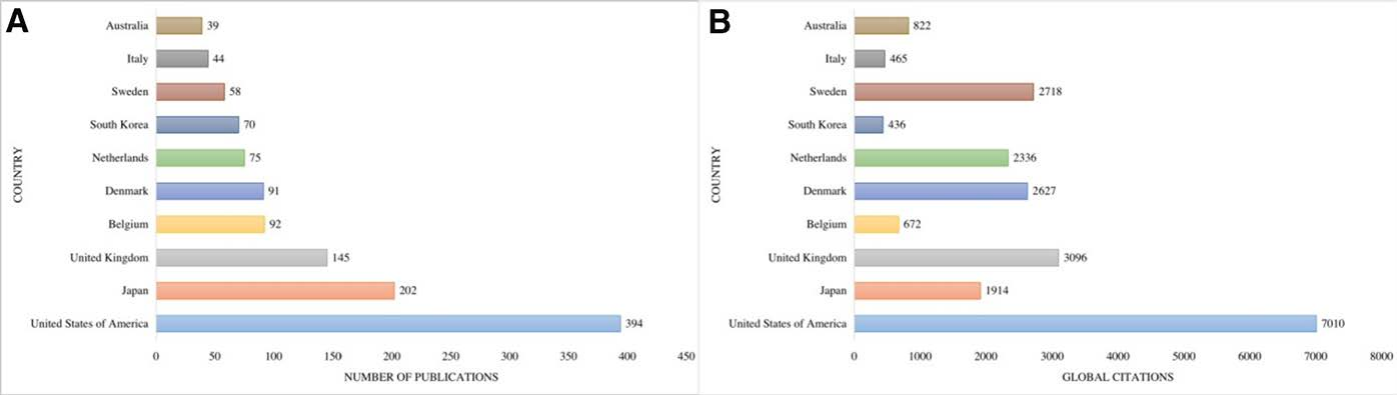
Top 10 leading countries in nocturia research.

**Figure 10.**
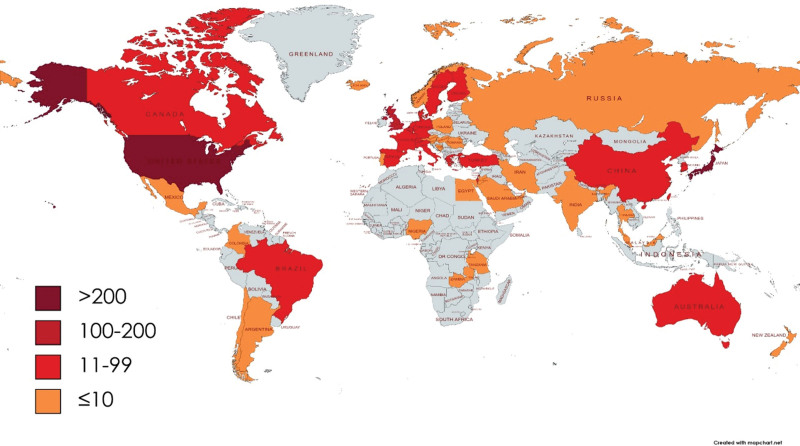
Heat mapping of countries involved in nocturia research. The values are representing the number of publications per country.

The initial search yielded 1479 documents. After applying the filtration options and exporting the data into the HistCite™ software, a total of 1445 documents were included in the final analysis. In total, 43.53% of documents were published as articles, followed by 34.67% meeting abstracts, 10.38% editorial materials, and 5.19% reviews, respectively, as shown in Figure 2. The top three most studied research areas in nocturia were urology nephrology, general internal medicine, and neuroscience neurology, as shown in Figure 3. The most productive year was 2019 (n = 121, citations = 335), while the number of publications on nocturia in 2021 was 52 (citations = 16) as of the searching date. The documents published in 2006 were cited 1458 times (n = 50), as shown in Figure 4. The top three most prolific authors in terms of the number of publications were Weiss JP (n = 97), followed by Everaert K (n = 73), and Johnson TM (n = 48), as shown in Figure 5. The most cited authors were Weiss JP (1658 citations) and Abrams P (1568 citations), as shown in Figure 5. The most attractive journals in nocturia research were the Journal of Urology (n = 293), Neuro-Urology and Urodynamics (n = 229), and BJU International (n = 98), as shown in Figure 6. In terms of citations, the most cited journals were Journal of Urology (3050 citations), BJU International (2679 citations), and Neuro-Urology and Urodynamics (1863 citations), as shown in Figure 6. The most widely used keywords in nocturia publications were nocturia (n = 1249), followed by nocturnal (n = 315), and patients (n = 291), as shown in Figure 7. The top three leading institutions in nocturia were Emory University, USA (n = 98), Ghent University Hospital Belgium (n = 69), and University of Pennsylvania, USA (n = 40), as shown in Figure 8. The leading countries in nocturia research were the USA (n = 394), followed by Japan (n = 282), and the United Kingdom (UK) (n = 145), as shown in Figures 9 and 10. In terms of citations, the USA was the top-cited country with 7010 citations, followed by the UK (citations = 3096) and Sweden (citations = 2718), as shown in Figure 9. The top 10 most-cited publications on nocturia, based on the number of citations, are presented in Table 1. The most cited publication was “Impact of Nocturia on Bone Fracture and Mortality in Older Individuals: A Japanese Longitudinal Cohort Study” published in the Journal of Urology in 2010 cited 150 times (12.50 citations per year).

**Table 1:** Top 10 publications in nocturia.

Ranking	Study	Global citations	Global citations per year
1	Nakagawa et al^[18]^	150	12.50
2	Van Kerrebroeck et al^[19]^	150	7.50
3	FitzGerald et al^[20]^	148	9.87
4	Bosch and Weiss^[21]^	144	12.00
5	Matthiesen et al^[22]^	143	5.50
6	Mathias et al^[23]^	127	3.53
7	Abraham et al^[24]^	126	7.00
8	Foley et al^[25]^	121	8.07
9	Lose et al^[26]^	115	6.05
10	Tikkinen et al^[27]^	114	8.77

### 3.1. Co-authorship countries network visualization

**Figure 11.**
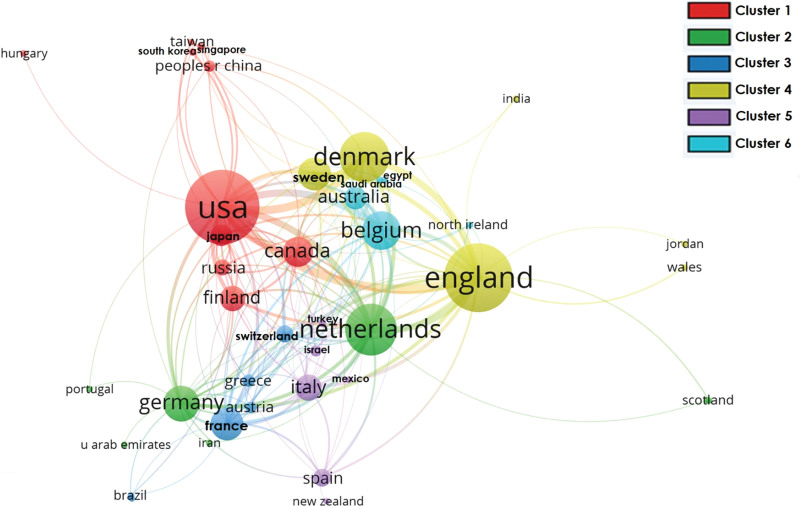
Co-authorship countries/regions network visualization.

Among the plotting countries, only four countries had a total link strength of over 100. The USA was the leading country with 256 total link strengths, followed by England with 225, the Netherlands with 148, and Denmark with 138. The co-authorship countries’ network visualization is presented in Figure 11.

### 3.2. Co-occurrence author keywords network visualization

**Figure 12.**
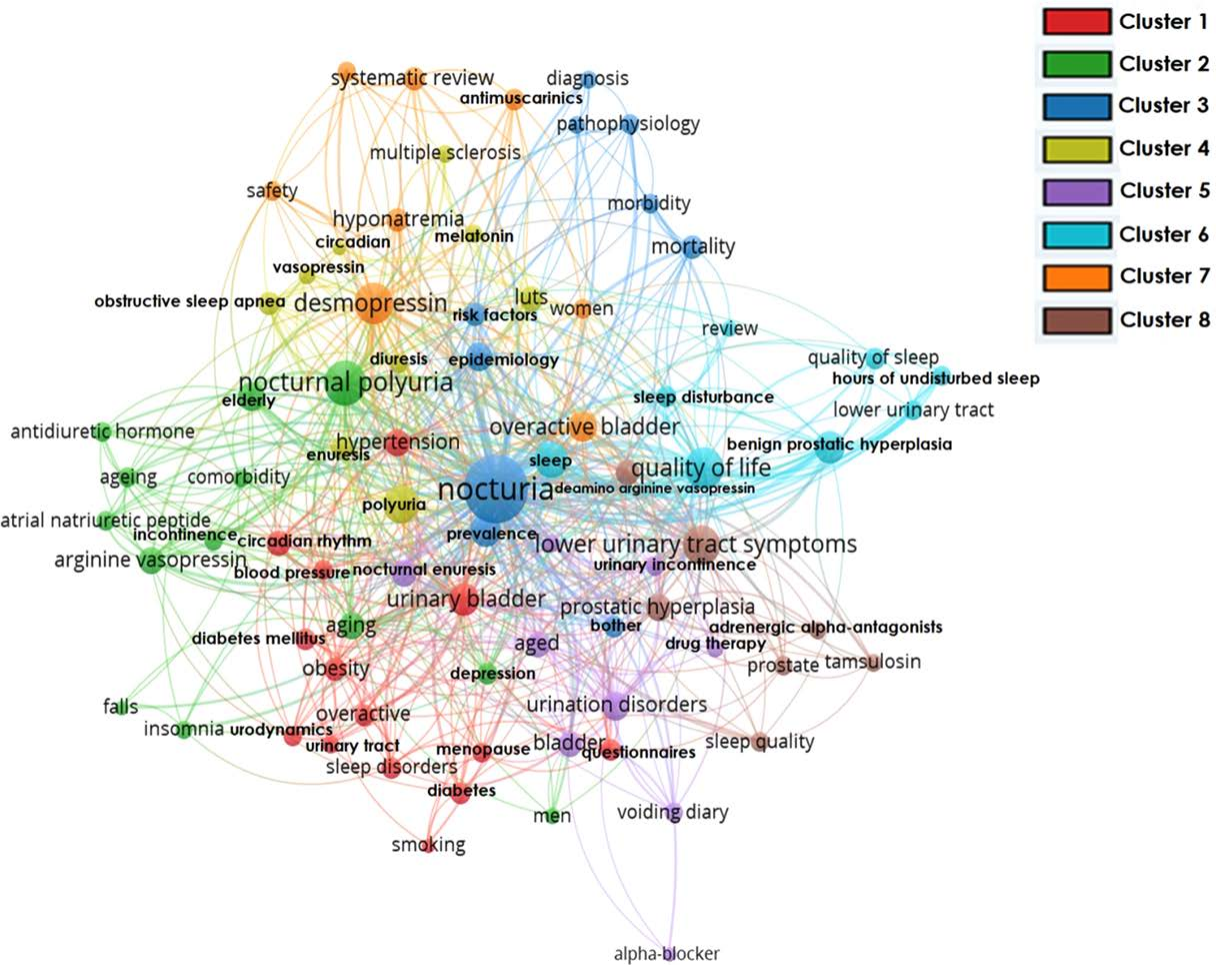
Co-occurrence author keyword network visualization.

Of the total author keywords, only 78 met the threshold and were plotted. The most significant and widely used author keywords were nocturia, with a total link strength of 926, and nocturnal polyuria 245. The network visualization mapping of author keywords is shown in Figure 12.

## 4. Discussion

To the best of our knowledge, this is the first bibliometric analysis to explore global research on nocturia indexed in the WoSCC database. Bibliometric studies play a significant role in providing referral points for researchers, policymakers, and medical practitioners. The study documented the most dynamic authors and countries, most frequent subject areas, most productive authors and journals, and citation patterns. There has been an increasing number of publications in the last two decades with more publications in 2019, owing to increasing awareness and research trends in the field.

Bibliometric studies are carried out to provide an overview of the published scientific literature, subjective and critical summarization of selected scientific studies/publications, and studies/publications with data that have more relevance than those subjective analyses.^[24]^ However, the bibliometric studies can be divided into three categories: evaluative techniques, review techniques, and relational techniques.^[25]^ Therefore, this type of study is vital to determine the trend related to a particular area of research and the support for future research funding.

The USA plays a leading role in publication and citation. Our study agrees with many other bibliometric studies^[26–30]^ in a different field, which confirms that the USA is a global leader in research-based publications and citations. This productivity is attributed to economic strength, availability, accessibility to research facilities, adequate funding, and strong collaboration with other institutes that carry research visibility and citation frequency. A similar trend was observed in Europe. In Nocturia research and citations, the USA is highest followed by Belgium. The publication trend does not represent the disease burden, but rather the priority of the researchers and government policies to focus on a specific health aspect and the allocation of funds for that.

It has been observed that all the articles were published in English and most of them were from the USA. It has also been observed that USA authors tend to cite publications from the USA, and reviewers from the USA tend to accept USA-based articles.^[31]^ Moreover, countries with a higher gross national product and those investing more in research and development tend to have higher output in biomedical research.^[32]^ These factors help us understand the publication and citation patterns of the literature on nocturia. Weiss JP from the USA has the highest number of citations among all authors in nocturia research. In his publications, he focused more on the solution to the problem rather than the diagnosis. He proposed certain treatments as well as the management of nocturia.^[33]^ This shows that the scientific literature offering the solution to a problem or treatment, or management of the disease is receiving more attention than the risk factor assessment and basic information about it in the case of nocturia.

Our results indicate that the publication time or age of the paper does not reflect the total number of citations of an article. An older article may have fewer citations, while the current article may receive more citations. The article discussing some of the important aspects of nocturia that was published in 2003 attracted 115 citations (6.05 citations per year), while another article published in 2010 attracted 150 citations (12.50 citations per year). While some of the articles become stagnant, after receiving a few citations, they have not received any new citations over the years. This is in line with citation dynamics, where a scientific article reaches its maximum citation rate three to ten years after publication and slowly declines afterward.^[34]^

Most of the articles among the top ten are on the association of nocturia with chronic and non-chronic diseases, while one is on the causes of nocturia. This reflects that most of the studies were based on data collected from patients via questionnaires or interviews. However, the scientific basis of the disease needs more attention to determine the proper cause, which will pave the way for treatment.

There was no association between the quality of a study and citations. This article may be positively or negatively cited by other authors. Citations only measure the impact of articles on the authors of other articles but do not reflect the impact on clinical practice or patient outcomes.^[35]^ Sometimes, authors may inflate the citations of an article by self-citation. This is a possibility, and the broad authorship in the current study indicates that this will not impact this study.

## 5. Conclusions

This study showed that there has been an increasing trend in publications on nocturia over the past few years. Urology nephrology is the most focused research area in nocturia. The USA was the most influential and highly contributing country in nocturia research. This study highlights important empirical evidence for researchers, clinicians, and physicians to understand research frontiers and trends, achievements, collaborative networks, and hotspot research topics in the research field of nocturia. It also provides insights for determining future research domains and seeking inter-collaborative cooperation to accelerate the efficiency of scientific research in nocturia management.

## 6. Limitations

The current study used a single database, the WoSCC database, for conducting this research. It is unique in terms of including older publications as compared to other databases such as Scopus. It provides broader transparency with coverage compared to other sources, such as Google Scholar. The use of Web of Science is beneficial as it outweighs potential limitations.

## Author contributions

Conceptualization: Tauseef Ahmad

Data curation: Tauseef Ahmad, Muhammad Khan, and Basem Al-Omari

Formal analysis: Tauseef Ahmad

Investigation: Tauseef Ahmad

Methodology: Tauseef Ahmad, Muhammad Khan, and Basem Al-Omari

Project administration: Tauseef Ahmad, and Basem Al-Omari.

Resources: Tauseef Ahmad

Software: Tauseef Ahmad

Validation: Tauseef Ahmad, Muhammad Khan, and Basem Al-Omari

Visualization: Tauseef Ahmad

Writing – original draft: Tauseef Ahmad

Writing – review & editing: Muhammad Khan and Basem Al-Omari
